# Differences in COVID-19 Outpatient Antiviral Treatment Among Adults Aged ≥65 Years by Age Group — National Patient-Centered Clinical Research Network, United States, April 2022–September 2023

**DOI:** 10.15585/mmwr.mm7339a3

**Published:** 2024-10-03

**Authors:** Claire M. Quinlan, Melisa M. Shah, Carol E. DeSantis, J. Bradford Bertumen, Christine Draper, Faraz S. Ahmad, Jonathan Arnold, Kenneth H. Mayer, Thomas W. Carton, Lindsay G. Cowell, Samantha Smith, Sharon Saydah, Jefferson M. Jones, Pragna Patel, Melissa Briggs Hagen, Jason Block, Emily H. Koumans

**Affiliations:** ^1^Coronavirus and Other Respiratory Viruses Division, National Center for Immunization and Respiratory Diseases, CDC; ^2^Harvard Medical School, Boston, Massachusetts; ^3^Inform and Disseminate Division, Office of Public Health Data, Surveillance, and Technology, CDC; ^4^Department of Population Medicine, Harvard Pilgrim Health Care Institute, Harvard Medical School, Boston, Massachusetts; ^5^Department of Medicine, Division of Cardiology, Northwestern University Feinberg School of Medicine, Chicago, Illinois; ^6^Department of Medicine, Division of General Internal Medicine, University of Pittsburgh School of Medicine, Pittsburgh, Pennsylvania; ^7^The Fenway Institute, Fenway Health, Boston, Massachusetts; ^8^Louisiana Public Health Institute, New Orleans, Louisiana; ^9^O’Donnell School of Public Health, UT Southwestern Medical Center, Dallas, Texas.

**Summary**

**What is already known about this topic?**

Older adults are at highest risk for hospitalization and death from COVID-19, with risk increasing with age. Outpatient antiviral treatment is effective at reducing these risks.

**What is added by this report?**

Fewer than one half of adults aged ≥65 years with an outpatient COVID-19 diagnosis received a recommended COVID-19 antiviral medication, including 48% among adults aged 65–74 years, 44% among those aged 75–89 years, and 35% among those aged ≥90 years. Among patients with severe outcomes, 21% had received an outpatient COVID-19 antiviral, compared with 47% of patients without severe outcomes.

**What are the implications for public health practice?**

Lower prevalence of outpatient antiviral treatment in the oldest age groups highlights the continued need to improve COVID-19 antiviral use by increasing awareness and testing, and facilitating early treatment in these groups.

## Abstract

Adults aged ≥65 years experience the highest risk for COVID-19–related hospitalization and death, with risk increasing with increasing age; outpatient antiviral treatment reduces the risk for these severe outcomes. Despite the proven benefit of COVID-19 antiviral treatment, information on differences in use among older adults with COVID-19 by age group is limited. Nonhospitalized patients aged ≥65 years with COVID-19 during April 2022–September 2023 were identified from the National Patient-Centered Clinical Research Network. Differences in use of antiviral treatment among patients aged 65–74, 75–89, and ≥90 years were assessed. Multivariable logistic regression was used to estimate the association between age and nonreceipt of antiviral treatment. Among 393,390 persons aged ≥65 years, 45.9% received outpatient COVID-19 antivirals, including 48.4%, 43.5%, and 35.2% among those aged 65–75, 76–89, and ≥90 years, respectively. Patients aged 75–89 and ≥90 years had 1.17 (95% CI = 1.15–1.19) and 1.54 (95% CI = 1.49–1.61) times the adjusted odds of being untreated, respectively, compared with those aged 65–74 years. Among 12,543 patients with severe outcomes, 2,648 (21.1%) had received an outpatient COVID-19 antiviral medication, compared with 177,874 (46.7%) of 380,847 patients without severe outcomes. Antiviral use is underutilized among adults ≥65 years; the oldest adults are least likely to receive treatment. To prevent COVID-19–associated morbidity and mortality, increased use of COVID-19 antiviral medications among older adults is needed.

## Introduction

One of the most important factors associated with increased risk for hospitalization and death among patients with COVID-19 is age ≥50 years, with risk increasing with increasing age (*1*–*4*). Adults aged ≥65 years accounted for approximately two thirds of all COVID-19–associated hospitalizations during October 2023–April 2024, with adults aged ≥75 years accounting for nearly one half of hospitalizations and in-hospital deaths (*1*). In 2022, the COVID-19–related mortality rate among persons aged 65–74 and ≥85 years was approximately 100 and 800 times as high, respectively, as that among persons aged 15–24 years (*4*). Despite the continued effectiveness of COVID-19 oral antiviral medications to prevent hospitalization and death (*3*), studies suggest low use among persons aged ≥65 years; however, less is known about the differences in use among older patients (*5*,*6*). To examine differences in treatment by age and other factors associated with treatment, such as underlying medical conditions, race, and ethnicity, electronic health record data from the National Patient-Centered Clinical Research Network (PCORnet)* were analyzed.

## Methods

### Study Population and Criteria for Inclusion or Exclusion

This cross-sectional study used electronic health record data from 28,053,928 adults aged ≥20 years at 28 U.S. health care systems participating in PCORnet, during April 2022–September 2023. Patients with SARS-CoV-2 infection (1,298,966) were identified using at least one of the following inclusion criteria: 1) laboratory-confirmed SARS-CoV-2 test result identified with Logical Observation Identifiers Names and Codes (LOINC)^†^; 2) an *International Classification of Diseases, Tenth Revision, Clinical Modification* (ICD-10-CM)^§^ diagnostic code for COVID-19 (U07.1 or U07.2); or 3) prescription or administration of an outpatient COVID-19 treatment (nirmatrelvir-ritonavir, molnupiravir, monoclonal antibody, or remdesivir).^¶^ The earliest COVID-19 diagnosis date by one of these three criteria was defined as the index date. Persons hospitalized on or before their index date (1,297,899) were excluded to limit the analysis to outpatient COVID-19 diagnoses; thus, 393,390 patients aged ≥65 years were selected for inclusion in the analysis.

### Population Characteristics and Outcome Definitions

Characteristics of patients aged 65–74, 75–89, and ≥90 years with COVID-19 were described by sex, race and ethnicity, area deprivation index (ADI),** underlying medical conditions, combined comorbidity index (CCI),^††^ use of immunosuppressive medication,^§§^ use of outpatient COVID-19 antivirals (overall and by medication) within 30 days of the index date, and outcome (*7*). Hospitalizations were inpatient encounters within 16 days of the index date. Severe outcome was defined as 1) hospitalization or 2) death or hospice (in-hospital death, out-of-hospital death, or discharge to hospice within 30 days of index date).^¶¶^

### Statistical Analysis

To compare differences by age, Pearson’s chi-square p-values were calculated. Because nearly all p-values were statistically significant at p<0.05, standardized mean differences (SMDs) were calculated among age groups to identify larger effect sizes; an SMD>0.2 was considered to represent large differences among groups.*** Logistic regression was used to estimate measures of association of age group with nontreatment, adjusting for sex, race, ethnicity, comorbidity index, and ADI score. To address a potential selection bias, a sensitivity analysis was conducted, excluding patients whose only index date was based on an antiviral prescription. All analyses were conducted using R software (version 4.2.3; R Foundation). This activity was reviewed by CDC, deemed not research, and was conducted consistent with applicable federal law and CDC policy.^†††^

## Results

### Population Characteristics, Outcomes, and Univariate Results by Age

Among 393,390 patients aged ≥65 years who received a COVID-19 diagnosis^§§§^ in an outpatient setting during April 2022–September 2023, a total of 221,798 (56.4%) were aged 65–74 years, 154,918 (39.4%) were aged 75–89 years, and 16,674 (4.2%) were aged ≥90 years (Table 1). Overall, 225,497 (57.3%) patients were women, 306,347 (80.7%) were non-Hispanic White, and 33,721 (8.9%) were non-Hispanic Black or African American. Among all 393,390 COVID-19 patients aged ≥65 years, 180,522 (45.9%) received outpatient antiviral treatment for COVID-19, 10,748 (2.7%) were hospitalized, and 2,422 (0.6%) died or were discharged to hospice. Statistically significant differences by age group were observed in the combined comorbidity score and several underlying medical conditions and treatment (Table 1). Prevalence of hospitalizations was 1.8% among patients aged 65–74 years, 3.5% among those aged 75–89 years, and 7.1% among those aged ≥90 years (SMD = 0.174) (Table 1) (Figure).

**TABLE 1:** Characteristics of persons aged 65–74, 75–89, and ≥90 years with an outpatient COVID-19 diagnosis — National Patient-Centered Clinical Research Network, United States, April 2022–September 2023

Characteristic	Age group, yrs no. (%)				SMD*
	All	65–74	75–89	≥90	
**Total**	**393,390 (100.0)**	**221,798 (56.4)**	**154,918 (39.4)**	**16,674 (4.2)**	**—**
**COVID-19 outpatient medication received within 30 days of the index date^†^**					
Any (nirmatrelvir-ritonavir, molnupiravir, monoclonal antibodies, or remdesivir)	**180,522 (45.9)**	107,320 (48.4)	67,338 (43.5)	5,864 (35.2)	0.180
Any oral (nirmatrelvir-ritonavir or molnupiravir)	**163,947 (41.7)**	99,863 (45.0)	59,420 (38.4)	4,664 (28.0)	0.239
Nirmatrelvir-ritonavir	**150,562 (38.3)**	93,149 (42.0)	53,441 (34.5)	3,972 (23.8)	0.262
Molnupiravir	**14,228 (3.6)**	7,190 (3.2)	6,295 (4.1)	743 (4.5)	0.042
Monoclonal antibodies	**12,316 (3.1)**	6,158 (2.8)	5,560 (3.6)	598 (3.6)	0.031
Remdesivir	**5,250 (1.3)**	1,725 (0.8)	2,839 (1.8)	686 (4.1)	0.148
**Defining index event^§^**					
COVID-19 diagnosis	**264,927 (67.3)**	149,333 (67.3)	104,252 (67.3)	11,342 (68.0)	0.010
COVID-19 laboratory test result (positive, detected, or presumptive)	**126,287 (32.1)**	65,975 (29.7)	53,598 (34.6)	6,714 (40.3)	0.148
COVID-19 medication prescription	**157,808 (40.1)**	94,912 (42.8)	57,996 (37.4)	4,900 (29.4)	0.188
COVID-19 medication administration	**4,133 (1.1)**	1,951 (0.9)	1,941 (1.3)	241 (1.4)	0.035
**Comorbidities^¶^**					
Cancer	**61,658 (15.7)**	31,352 (14.1)	27,914 (18.0)	2,392 (14.3)	0.071
Cardiac	**264,646 (67.3)**	137,878 (62.2)	113,473 (73.2)	13,295 (79.7)	0.262
HIV	**1,264 (0.3)**	1,028 (0.5)	228 (0.1)	8 (0.0)	0.057
Immunodeficiency	**84,969 (21.6)**	49,794 (22.5)	32,608 (21.0)	2,567 (15.4)	0.121
Kidney disease	**71,081 (18.1)**	29,689 (13.4)	35,782 (23.1)	5,610 (33.6)	0.327
Liver	**7,623 (1.9)**	5,298 (2.4)	2,192 (1.4)	133 (0.8)	0.086
Metabolic	**151,598 (38.5)**	89,273 (40.2)	57,745 (37.3)	4,580 (27.5)	0.181
Neurologic	**24,973 (6.3)**	7,255 (3.3)	14,204 (9.2)	3,514 (21.1)	0.383
Psychiatric and substance abuse	**79,769 (20.3)**	46,959 (21.2)	29,871 (19.3)	2,939 (17.6)	0.060
Pulmonary	**77,037 (19.6)**	43,331 (19.5)	30,850 (19.9)	2,856 (17.1)	0.048
Other	**4,203 (1.1)**	2,374 (1.1)	1,659 (1.1)	170 (1.0)	0.003
None of above comorbidities	**69,758 (17.7)**	43,687 (19.7)	23,802 (15.4)	2,269 (13.6)	0.109
Combined comorbidity index score**					
<0	**57,462 (15.0)**	36,530 (16.8)	19,528 (13.0)	1,404 (8.7)	0.484
0	**126,700 (33.0)**	80,988 (37.3)	42,637 (28.3)	3,075 (19.1)	
1	**71,240 (18.6)**	42,146 (19.4)	26,906 (17.9)	2,188 (13.6)	
2	**41,990 (10.9)**	21,425 (9.9)	18,420 (12.2)	2,145 (13.3)	
3	**26,342 (6.9)**	11,835 (5.5)	12,763 (8.5)	1,744 (10.8)	
4	**17,124 (4.5)**	7,078 (3.3)	8,602 (5.7)	1,444 (9.0)	
5	**12,457 (3.2)**	4,934 (2.3)	6,392 (4.2)	1,131 (7.0)	
>5	**30,515 (8.0)**	12,111 (5.6)	15,461 (10.3)	2,943 (18.3)	
Missing	**9,560 (—)**	4,751 (—)	4,209 (—)	600 (—)	
**Immunosuppressive medication within year of index event**					
Corticosteroid (one or more event)	**58,931 (15.0)**	34,667 (15.6)	22,493 (14.5)	1,771 (10.6)	0.099
Immunosuppressive medication (one or more event)	**13,250 (3.4)**	8,767 (4.0)	4,285 (2.8)	198 (1.2)	0.118
**Sex**					
Women	**225,497 (57.3)**	127,722 (57.6)	87,074 (56.2)	10,701 (64.2)	0.109
Men	**167,869 (42.7)**	94,060 (42.4)	67,836 (43.8)	5,973 (35.8)	
**Race and ethnicity^††^**					
AI/AN	**1,436 (0.4)**	949 (0.4)	464 (0.3)	23 (0.1)	0.110
Asian	**9,800 (2.6)**	5,514 (2.6)	3,775 (2.5)	511 (3.2)	
Black or African American	**33,721 (8.9)**	21,764 (10.2)	10,883 (7.3)	1,074 (6.7)	
NH/PI	**309 (0.1)**	195 (0.1)	104 (0.1)	10 (0.1)	
White	**306,347 (80.7)**	168,794 (78.9)	124,306 (83.1)	13,247 (83.1)	
Multiple races or other races	**8,050 (2.1)**	4,702 (2.2)	3,005 (2.0)	343 (2.2)	
Hispanic or Latino	**19,924 (5.2)**	12,128 (5.7)	7,055 (4.7)	741 (4.6)	
Missing	**13,803 (—)**	7,752 (—)	5,326 (—)	725 (—)	
**COVID-19 vaccination history, no. of recorded doses^§§^**					
1	**23,620 (6.0)**	13,948 (6.3)	8,783 (5.7)	889 (5.3)	0.114
2	**55,441 (14.1)**	30,314 (13.7)	22,766 (14.7)	2,361 (14.2)	
3	**71,279 (18.1)**	41,103 (18.5)	27,595 (17.8)	2,581 (15.5)	
≥4	**73,512 (18.7)**	40,706 (18.4)	30,188 (19.5)	2,618 (15.7)	
0 or missing	**169,538 (43.1)**	95,727 (43.2)	65,586 (42.3)	8,225 (49.3)	
**Received ≥1 COVID-19 vaccine dose in the previous 6 months**	**73,595 (18.7)**	41,768 (18.8)	29,152 (18.8)	2,595 (15.6)	0.058
**Area deprivation index^¶¶^**					
Q1	**116,910 (35.4)**	64,827 (34.7)	47,074 (36.2)	5,009 (36.8)	0.050
Q2	**85,519 (25.9)**	47,702 (25.5)	34,248 (26.3)	3,569 (26.2)	
Q3	**73,275 (22.2)**	41,562 (22.3)	28,850 (22.2)	2,863 (21.0)	
Q4	**54,892 (16.6)**	32,695 (17.5)	20,016 (15.4)	2,181 (16.0)	
Missing	**62,794 (—)**	35,012 (—)	24,730 (—)	3,052 (—)	
**Hospitalization**					
Inpatient encounter 1–16 days after index event	**10,748 (2.7)**	4,087 (1.8)	5,471 (3.5)	1,190 (7.1)	0.174
No. of days after index event to hospitalization, mean (SD)	**4.50 (4.49)**	4.98 (4.70)	4.34 (4.41)	3.62 (3.89)	0.209
**Death or discharge to hospice**					
0–30 days after index event	**2,422 (0.6)**	623 (0.3)	1,309 (0.8)	490 (2.9)	0.147
No. of days after index, mean (SD)	**11.82 (8.88)**	12.00 (9.27)	11.90 (8.77)	11.38 (8.68)	0.047
No. of patients with hospitalization or death or discharge to hospice***	**12,543**	4,530	6,449	1,564	—
With evidence of any outpatient treatment	**2,648 (21.1)**	933 (20.6)	1,372 (21.3)	343 (21.9)	0.022
With evidence of oral antiviral treatment	**1,808 (14.4)**	621 (13.7)	821 (12.7)	193 (12.3)	0.027
Without evidence of outpatient treatment	**9,895 (78.9)**	3,597 (79.4)	5,077 (78.7)	1,221 (78.1)	0.022
**No. of patients with no hospitalization and no death or discharge to hospice**	**380,847**	217,268	148,469	15,110	—
With evidence of any outpatient treatment	**177,874 (46.7)**	106,387 (49.0)	65,966 (44.4)	5,521 (36.5)	0.168
With evidence of oral antiviral treatment	**157,726 (41.4)**	99,242 (45.7)	58,599 (39.5)	4,471 (29.6)	0.224
Without evidence of outpatient treatment	**202,973 (53.3)**	110,881 (51.0)	82,503 (55.6)	9,589 (63.5)	0.168

**Abbreviations:** ADI = area deprivation index; AI/AN = American Indian or Alaska Native; BMI = body mass index; NH/PI = Native Hawaiian or Pacific Islander; Q = quartile; SMD = standardized mean difference.

* SMD values are reported because all p-values were significant at p<0.05, except for mean number of days from hospitalization to death or discharge to hospice (p = 0.453), COVID-19 diagnosis as index (p = 0.159), “Other” comorbidities (p = 0.821), and all values in hospitalized/died/discharge to hospice treatment status. SMDs were calculated for each characteristic or condition compared with not having that characteristic or condition. For characteristics with categories that are mutually exclusive (combined comorbidity index score, sex, race and ethnicity, COVID-19 vaccination history, number of recorded doses, and ADI) the SMD measured the effect size of each age group and category.

^†^ A total of 1,818 (0.05%) patients aged ≥65 years with COVID-19 received more than one outpatient antiviral or monoclonal antibody, including 16 persons who received three different treatments.

^§^ Participants could have more than one qualifying index event; therefore, these categories are not mutually exclusive.

^¶^ At least one record in the 3 years preceding index date: Cardiac = cerebrovascular disease, congestive heart failure, arrythmia, coronary artery disease, hypertension, or peripheral vascular disease; Metabolic = type 1 diabetes, type 2 diabetes, overweight (BMI 25–30 kg/m2), obesity (BMI ≥30 kg/m^2^), or severe obesity (BMI ≥40 kg/m^2^); Pulmonary = alpha-1 antitrypsin deficiency, bronchopulmonary dysplasia, chronic pulmonary disorder, asthma, chronic obstructive pulmonary disease, cystic fibrosis, or pulmonary circulation disorder; Kidney disease = chronic kidney disease, or end stage renal disease with dialysis; Immunodeficiency = primary immunodeficiency, inflammatory bowel disease, systemic lupus erythematosus, solid organ or stem cell transplant, rheumatoid arthritis, cancer, or multiple sclerosis;. Liver = cirrhosis, hepatitis B, or hepatitis C; Neurologic = dementia, seizure or epilepsy disorder, Parkinson disease, or hemiplegia; Psychiatric and substance abuse = alcohol abuse, smoking, substance use disorder, or mental health disorder; and Other = anemia, autism, coagulopathy, Down syndrome, or inactivity.

** Categories were generated from the combined comorbidity index score.

^††^ Persons of Hispanic or Latino (Hispanic) origin might be of any race but are categorized as Hispanic; all racial groups are non-Hispanic. The “Other” category includes persons of multiple racial and ethnicity groups.

^§§^ Vaccination data includes any COVID-19 vaccination before the index date.

^¶¶^ Patient 5-digit zip codes were mapped to socioeconomic status by normalized (ADI) value (0–100). Lower values are associated with lower deprivation, and higher values are associated with higher deprivation. For this mapping, values were grouped into quartiles using the count of zip codes. Q1 represents the lowest range of ADI values, and Q4 represents the highest range of ADI values (Q1 = 0–38; Q2 = 39–43; Q3 = 44–49; and Q4 = 50–100). Additional ADI index information is available at https://www.neighborhoodatlas.medicine.wisc.edu/.

*** Hospitalization within 16 days of index diagnosis, or death or discharge to hospice within 30 days of index diagnosis.

**FIGURE:**
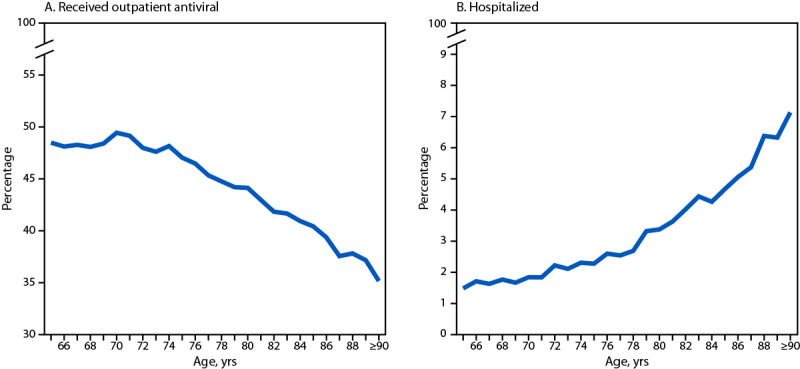
Percentage of adults aged ≥65 years with COVID-19 who received an outpatient antiviral medication* (A) and who were hospitalized^†^ (B), by age — National Patient-Centered Clinical Research Network, United States, April 2022–September 2023 * Patients with SARS-CoV-2 infection were identified using at least one of the following inclusion criteria: 1) laboratory-confirmed positive SARS-CoV-2 test result identified with Logical Observation Identifiers Names and Codes (LOINC); 2) an *International Classification of Diseases, Tenth Revision, Clinical Modification* (ICD-10-CM) diagnostic code for COVID-19 (U07.1 or U07.2); or 3) prescription or administration of an outpatient COVID-19 treatment (nirmatrelvir-ritonavir, molnupiravir, monoclonal antibody, or remdesivir). The earliest COVID-19 infection diagnosis date by one of these three criteria was defined as the index date. ^†^ Hospitalizations were inpatient encounters within 16 days of the index date.

### Differences in Receipt of Antiviral Treatment by Age

Receipt of outpatient antiviral treatment^¶¶¶^ varied across age groups: 48.4% among patients aged 65–74 years, 43.5% among those aged 75–89 years, and 35.2% among those aged ≥90 years received a COVID-19 antiviral (SMD = 0.180) (Figure). Among patients aged 65–74 years, 45.0% received an oral antiviral medication compared with 38.4% among those aged 75–89 years and 28.0% among those aged ≥90 years**** (SMD = 0.239). The percentage of patients aged ≥90 years who were treated with molnupiravir (4.5%) or intravenous remdesivir (4.1%) was higher than the percentage of those aged 65–74 years who received these medications (3.2% and 0.8%, respectively). Compared with COVID-19 patients aged 65–74 years, the adjusted odds ratio (aOR) for not being treated with outpatient antiviral medications for COVID-19 was 1.17 among patients aged 75–89 years and 1.54 among those aged ≥90 years. Compared with those with a CCI score <1 (lower mortality risk), those with a CCI score? of 1–2 and ≥3 had increased odds of not receiving antiviral treatment (aOR = 1.09 and 1.47, respectively) (Table 2). Similar results were found when 62,910 patients whose index date was based on a prescription only were excluded. Among 12,543 patients with severe outcomes, 2,648 (21.1%) had received an outpatient COVID-19 antiviral, compared with 177,874 (46.7%) of 380,847 patients without severe outcomes (Table 1).

**TABLE 2:** Association of age and combined comorbidity index with odds of not receiving outpatient COVID-19 antiviral medication*^,†,§^ — National Patient-Centered Clinical Research Network, United States, April 2022–September 2023

Characteristic	Odds ratio (95% CI)	
	Unadjusted	Adjusted
**Age group, yrs**		
65–74	Ref	Ref
75–89	1.22 (1.20–1.24)	1.17 (1.15–1.19)
≥90	1.73 (1.67–1.79)	1.54 (1.49–1.61)
**Combined comorbidity index** ^¶^		
≤0	Ref	Ref
1–2	1.09 (1.08–1.11)	1.09 (1.07–1.10)
≥3	1.58 (1.55–1.61)	1.47 (1.44–1.50)

**Abbreviation: **Ref = referent group.

* Nirmatrelvir-ritonavir, molnupiravir, monoclonal antibody, or remdesivir.

^†^ Adjusted for sex, race and ethnicity, and area deprivation index.

^§^ Model sample size = 312,003 (cohort size = 393,390). Missing values: race = 3.5%; sex <0.01%; combined comorbidity index = 2.4%; area deprivation index = 16.0%.

^¶^ Combined comorbidity index is a validated score for the Medicare population used to predict 1-year mortality and ranges from −2 to 265. Higher values are associated with increased mortality risk.

## Discussion

This analysis of nearly 400,000 COVID-19 patients aged ≥65 years found that fewer than one half received outpatient COVID-19 antiviral treatment. This finding is consistent with other studies: among patients aged 65–79 years and ≥80 years, one reported 37% and 9% antiviral use (*3*) and another reported 39.9% and 30.7% use, respectively (*6*). This study found that prevalence of receipt of antivirals decreased progressively and substantially with increasing age in persons aged 65 to ≥90 years, after controlling for the number of underlying medical conditions and other demographic factors.

Several real-world studies, including those conducted since the emergence of SARS-CoV-2 Omicron variant in January 2022, have demonstrated that COVID-19 antivirals are effective in preventing hospitalization and death (*3*). Because older age is a strong risk factor for severe COVID-19–associated outcomes, and COVID-19 hospitalizations continue to disproportionately affect older patients (*1*–*4*), treatment of COVID-19, including cases in older adults, is critical to the prevention of severe outcomes.

Among older patients, frequent self-reported reasons for nonuse of antivirals include the presence of mild signs and symptoms, lack of awareness of eligibility, and absence of a provider recommendation (*8*). Other potential barriers to treatment among older patients include delays in seeking treatment after symptom onset and missing the treatment window (5–7 days after symptom onset) (*8*). Challenges to COVID-19 antiviral use include obtaining testing (*9*), acquiring an antiviral prescription after receiving a positive SARS-CoV-2 test result, and accessing treatment, with each step potentially requiring a separate visit to a health care facility.

Older age is associated with increasing numbers of comorbidities and potentially related medications, which might lead to patient and provider hesitation to commence treatment, based on concerns about drug interactions with nirmatrelvir-ritonavir or contraindications among patients with severe hepatic and renal disease. Lower antiviral use prevalence among persons who have more underlying medical conditions might be consistent with concern about drug interactions or difficulty in temporarily discontinuing or adjusting other concomitantly prescribed medications in older persons. However, absolute contraindications are unlikely to be the only reason for this finding because the decrease in antiviral use prevalence by age persisted even after controlling for CCI score. In addition, the use of the well-tolerated oral molnupiravir and intravenous remdesivir, which have few medication contraindications, increased only slightly with increasing age, and this analysis suggests that these medications have not closed the age-related treatment gap. Older adults and their providers might also have concerns about possible rebound after treatment, including the need to isolate should symptoms recur, although a review found similar frequencies of viral rebound among persons who received or did not receive treatment for COVID-19 (*10*).

### Limitations

The findings in this report are subject to at least four limitations. First, this study excluded patients who received a COVID-19 diagnosis upon hospital admission, although the trend of decreasing oral antiviral use with age was similar when those diagnosed with COVID-19 during hospitalization were included. Second, selection bias might have resulted from exclusion of persons who did not have electronic health record documentation of receipt of a positive test result or treatment for COVID-19; this exclusion might also vary by age, possibly underestimating the prevalence of COVID-19 or treatment. Third, this data asset represents predominantly urban-based health care systems that identified a small portion of laboratory- or provider-confirmed cases during April 2022–September 2023; as such, the results might have overestimated overall receipt of treatment, but the effect by age is unclear. Finally, although few contraindications to receiving antivirals exist, this study could not exclude persons with contraindications to nirmatrelvir-ritonavir, possibly underestimating the prevalence of treatment. However, the effect of contraindications by age is unclear.

### Implications for Public Health Practice

In this study of older adult patients with laboratory-confirmed COVID-19, antiviral treatment was underutilized, particularly among the oldest adults, who are at highest risk for severe outcomes. These findings highlight the importance of prioritizing public health efforts to improve antiviral use among older adults with COVID-19, particularly those aged ≥75 years. In addition to vaccination and access to early sensitive diagnostics such as polymerase chain reaction testing, COVID-19 treatment should be routinely discussed with older adults with mild or moderate COVID-19. Among eligible persons, antiviral treatment should be started within 5–7 days of symptom onset. Public health efforts to address provider hesitancy and patient knowledge of COVID-19 antivirals and to eliminate barriers to COVID-19 diagnostics and treatment are needed, especially among older adults.

## Footnotes

* PCORnet is a national network that facilitates access to health care data and interoperability through the use of a common data model across participating health care systems. The PCORnet Common Data Model includes demographic characteristics, diagnoses, prescriptions, procedures, and laboratory test results, among other inpatient and outpatient elements, from approximately 30 million patients. https://pcornet.org/data

† LOINC is a code system that includes identifiers, names, and codes for clinical and laboratory observations, health care screening instruments, and document types. https://loinc.org

§
https://www.cdc.gov/nchs/icd/icd-10-cm/files.html

¶ Nirmatrelvir-ritonavir and molnupiravir are taken orally; remdesivir and monoclonal antibodies are administered intravenously. The only monoclonal antibody available for outpatient treatment during the study period was bebtelovimab. However, on November 30, 2022, the Food and Drug Administration announced that bebtelovimab was not authorized because it was not expected to neutralize the variants in circulation at that time.

** ADI is a mapping tool that displays relative socioeconomic conditions of a neighborhood. Patients’ 5-digit zip codes are mapped to socioeconomic status by normalized ADI value (0–100). Lower values are associated with lower deprivation and higher values with higher deprivation. Values are grouped into quartiles using the count of zip codes. Quartile 1 represents the lowest range and quartile 4 the highest range of ADI values (quartile 1 = 0–38; quartile 2 = 39–43; quartile 3 = 44–49; and quartile 4 = 50–100). https://www.neighborhoodatlas.medicine.wisc.edu

†† CCI score is a validated numerical value for the Medicare population used to predict 1-year mortality and ranges from −2 to 265. Higher values are associated with increased mortality risk.

§§ The use of immunosuppressive medications was defined as having a prescription for an immunosuppressing medication or corticosteroids (parenteral or oral) at least once during the 365 days before the index COVID-19 diagnosis.

¶¶ This outcome is determined only from data recorded in an electronic health record. PCORnet data identifies in-facility deaths but not out-of-facility deaths unless linked to a death or vital records registry.

*** SMDs were calculated by averaging all the pairwise absolute differences among the three groups scaled by their SDs.

††† 45 C.F.R. part 46, 21 C.F.R. part 56; 42 U.S.C. Sect. 241(d); 5 U.S.C. Sect. 552a; 44 U.S.C. Sect. 3501 et seq.

§§§ Among 393,390 patients aged ≥65 years with COVID-19, 67% had a COVID-19 diagnostic code, 32.1% had received a laboratory-confirmed positive SARS-CoV-2 test result, 40.1% were prescribed an outpatient COVID-19 treatment, and 1.1% were administered an outpatient COVID-19 treatment. Patients could have met more than one of these criteria.

¶¶¶ A total of 1,818 (0.05%) patients aged ≥65 years with COVID-19 received more than one outpatient antiviral or monoclonal antibody, including 16 persons who received three different medications.

**** In this data asset, 80% of persons hospitalized received a COVID-19 diagnosis on or after their day of admission and were excluded from this analysis. Decreased oral antiviral use in older age groups when hospitalized patients were included was observed (42.1% among those aged 65–74 years, 33.6% among those aged 75–89 years, and 21.7% among those aged ≥90 years).
